# Effect of Granzyme K, FasL and Interferon-γ Expression in Placentas with Preeclampsia

**DOI:** 10.3390/biomedicines12040842

**Published:** 2024-04-11

**Authors:** Martina Vukoja, Marina Ćurlin, Katarina Vukojević, Nevenka Jelić-Knezović, Anita Kolobarić, Martina Orlović Vlaho, Violeta Šoljić

**Affiliations:** 1 Department of Histology and Embryology, School of Medicine, University of Mostar, Bijeli Brijeg bb, 88000 Mostar, Bosnia and Herzegovina; martina.vukoja@mef.sum.ba (M.V.); anita.kozul@mef.sum.ba (A.K.); violeta.soljic@mef.sum.ba (V.Š.); 2 Faculty of Health Studies, University of Mostar, Bijeli Brijeg bb, 88000 Mostar, Bosnia and Herzegovina; marina.curlin@fzs.sum.ba (M.Ć.); martina.vlaho@fzs.sum.ba (M.O.V.); 3 Center for Translational Research in Biomedicine, University of Split School of Medicine, Šoltanska 2, 21000 Split, Croatia; 4 School of Medicine, University of Mostar, Bijeli Brijeg bb, 88000 Mostar, Bosnia and Herzegovina; nevenka.jelic@mef.sum.ba; 5 Department of Gynecology, University Hospital Mostar, Bijeli Brijeg bb, 88000 Mostar, Bosnia and Herzegovina

**Keywords:** trophoblast markers, preeclampsia, granzyme K, FasL, interferon-γ

## Abstract

This study aimed to investigate the cytotoxic activity of decidual lymphocytes and the mRNA/protein expression of cytotoxic proteins in various cell types in the context of preeclampsia (PE) compared to those of healthy pregnancies. We analyzed fresh decidua basalis tissue and tissue embedded in paraffin (FFPE) from PE pregnancies (n = 15) and compared them with those of healthy pregnancies (n = 15) of the corresponding gestational age. Using double immunofluorescence staining, we observed differences in the intensity and distribution of staining for granzyme K (GZMK) and FasL in extravillous trophoblasts. RT-qPCR analysis of FFPE placental tissue showed that GZMK mRNA expression was statistically higher (*p* < 0.0001) in PE compared to that of healthy controls. On the contrary, there was a low expression (*p* < 0.001) of FasL mRNA in PE compared to controls, while there was no statistically significant difference for IFN-γ mRNA between PE and controls. Although the level of cytotoxic activity changed depending on the ratio of effector and target cells, there was no significant difference observed between PE and controls in this in vitro study. In conclusion, in PE, extravillous trophoblasts exhibited increased expression of GZMK and decreased expression of FasL. These changes may contribute to impaired trophoblast invasion. However, these alterations did not appear to affect the cytotoxic properties of decidual lymphocytes. Additionally, the possibility of cell sorter separation of decidual lymphocytes would greatly contribute to a better understanding of single cells’ genetic profiles.

## 1. Introduction

The human placenta is composed of two types of trophoblasts: villous and extravillous trophoblasts. The villous trophoblast (VT) facilitates gas and nutrient transfer, whereas extravillous trophoblasts (EVTs) penetrate and alter the uterine wall and blood vessels to provide maternal blood to the developing fetus. Anomalous trophoblast differentiation leads to placental dysfunction and is linked to a range of pregnancy issues such as preeclampsia (PE) and fetal growth restriction. [1]. During the first trimester of pregnancy, the trophoblast plays a key role in the angiogenic function, especially in attacks on the mother’s myometrial spiral artery. Therefore, the disruption of trophoblast functions can result in a series of adverse pregnancy outcomes such as fetal growth restriction, spontaneous abortion, stillbirth and PE [2]. Preeclampsia is a complex multisystemic disease that includes angiogenic, metabolic, immunological and genetic factors. It is diagnosed with sudden hypertension after the 20th week of gestation with the appearance of proteinuria, dysfunction of maternal organs or dysfunction of the placenta [3,4].

The most invasive cells of the placenta are EVTs, which are in direct communication with the immune cells of the maternal decidua [1,4,5,6]. However, the complex interactions between immune cells and trophoblasts at the maternal–fetal interface are still largely unknown. IFN-γ is a key cytokine produced by NK cells and T-lymphocytes. It has been implicated in regulating trophoblast invasion and immune tolerance during pregnancy [7]. According to recent research, IFN-γ can increase EVT apoptosis and inhibit the migration and invasion of EVT cells, thus avoiding excessive EVT invasion and reducing PE [8]. Due to the contradictory results on IFN-γ, Yang et al. showed that the levels of IFN-γ in mothers with PE were higher than those in healthy pregnant women [9].

NK cells play a critical role in the immune response by eliminating target cells through cytotoxicity and secreting cytokines to modulate immune function. The mechanism of target cell recognition involves a balance of activating and inhibitory signals, with NK cells primarily targeting cells that lack MHC-I expression or express stress-induced ligands [10]. Immunoglobulin-like transcript (ILT) receptors represent an important group of receptors involved in the regulation of NK cell activity, particularly in the context of pregnancy. The interaction between HLA-G and ILT receptors represents an important immunomodulatory mechanism at the maternal–fetal interface, contributing to the establishment and maintenance of immune tolerance during pregnancy [11]. Genetic studies based on animal models have highlighted the importance of decidual NK cells in spiral artery remodeling, trophoblast invasion and placental development. Dysregulation of these processes, including altered IFN-γ levels, may contribute to the pathogenesis of pregnancy complications such as preeclampsia [12]. 

With gestational aging, the total number of decidual lymphocytes increases, with CD8 + T cells being the most predominant. The majority of CD8 + T cells in the decidual tissue are activated effector memory T cells, whose role comprises the high expression of cytolytic molecules. It was observed that the proportion of memory CD8 + T cells was reduced in pregnant women with PE compared to that in healthy pregnancies of the same gestational age [4,13]. FasL is a ligand expressed on the surface of effector lymphocytes, including NK and T cells, that binds to Fas and initiates apoptosis. FasL is stored in the cytoplasmic granules of CD8 + T cells and NK cells [14]. The Fas/FasL system is responsible for the initiation of apoptosis in various tissues since *Fas/FasL* genes play a significant role in the homeostasis of the immune response and the regulation of the apoptotic pathway [15,16,17]. In the villous part of the placenta, apoptotic mechanisms are of great importance in regulating the growth of the placenta during a healthy pregnancy. The increased level of apoptosis of villous trophoblasts of the placenta is closely related to PE [18,19]. 

Granzyme K (GZMK) is a member of the serine protease family similar to granzyme A; it induces rapid, caspase-independent cell death [20]. GZMK is stored in granules inside cytotoxic cells of the immune system, such as NK cells and cytotoxic T cells. While GZMK is traditionally associated with cytotoxic functions in NK cells and cytotoxic T cells, recent research has revealed its diverse roles in regulating immune responses, inflammation, immune tolerance and lymphocyte migration [21]. However, GZMK was not investigated in PE. Our previous study found the expression of cytotoxic proteins, including GZMA and GZMB, was decreased in decidual CD8 + T cells in preeclampsia [4].

The impact of effector memory CD8 + T and NK cells on preeclampsia is largely investigated. Namely, effector memory CD8 T cells play a crucial role in the immune response during pregnancy, as they help maintain a balance between the immune tolerance of the feto-placental unit and the response to infections. On the other hand, decidual NK cells are poorly cytolytic and release cytokines/chemokines that induce trophoblast invasion. There is a gap in the existing literature about the involvement of cytotoxic lymphocytes in the maternal part of the placenta during PE, which would clarify the consequence of PE on the immunological aspect of the placenta. Therefore, the aim of this study is to examine whether there is a correlation between changes in the mRNA and protein expression of GZMK, FasL and IFN-γ in decidual CD8 + T cells and NK cells as well as the cytotoxic activity of decidual lymphocyte cells.

## 2. Materials and Methods

### 2.1. Patient Data

PE was defined in accordance with the guidelines by the American College of Obstetricians and Gynecologists (ACOG). Briefly, it is defined through hypertension with higher systolic (≥140 mmHg) and a higher diastolic (≥90 mmHg) pressure. If proteinuria is absent, PE can be defined with one or more conditions such as liver damage, progressive renal failure, pulmonary edema, thrombocytopenia and cerebral and visual impairment. Systolic and diastolic pressure was measured twice within 4 h in women with previously normal blood pressure. The control group comprised placentas from normal pregnancy. All placenta samples were gestational age-matched (control vs. PE placenta). 

The data on the mother risk factors and fetus risk factors and from PE and the control were observed as follows: systolic and diastolic blood pressure in pregnancy; gestational age; maternal age; body mass index (BMI) before pregnancy; intrauterine growth restriction (IUGR) in the current pregnancy; birth weight and postpartum complications; and cesarean section or vaginal delivery. Excluding criteria for PE and the control group were diabetes mellitus type 1 or 2, chronic hypertension, any inflammatory disease, chorioamnionitis, multiple gestations and assisted reproduction methods. In total, 30 placentas were included in the study (15 placenta samples were preeclampsia and 15 placenta samples were normal healthy pregnancies) (Table 1).

### 2.2. Tissue Processing

Placental tissues were collected after C-section or after vaginal deliveries from the Department of Gynecology and Obstetrics University Hospital Center Mostar from 2020 to 2023. The study was approved by the Ethics committee of University Hospital Mostar (protocol code 406/19 and date of approval 24 February 2019) according to the guidelines of the Declaration of Helsinki. Placentas were promptly retrieved from the delivery room within 20 min of birth. They underwent fixation in 4% formalin before being dispatched to the Department for further analysis. Following a macroscopic examination, samples were extracted from the central region of the placental disk, according to if there were any visible signs indicative of preeclampsia, using established protocols. These placental sections were then fully embedded in paraffin, as outlined in the prior literature [4]. 

### 2.3. Double Immunofluorescence

Placental tissue samples containing basal decidua were cut, washed in phosphate buffer (PBS), dehydrated in ethanol, purified in xylen and embedded in paraffin. Tissue sections were cut (4 μm) on a rotatory microtome and mounted on silanized glass slides, deparaffinized in xylen, rehydrated through descending concentrations of ethanol and washed in distilled water. Four antigen retrieval slides were incubated in citrate buffer at pH 6 for 15 min in a microwave oven. After this step, antigen retrieval sections were incubated with a combination of primary antibodies (Table 2) for 1 h. After incubation, the primary antibody sections were washed in PBS and incubated with an appropriate combination of secondary antibodies: anti-rabbit IgG (H + L) and F(ab’)2 Fragment Alexa Fluor® 594 Conjugate (8889as; Cell Signaling, Boston, MA, USA) diluted 1:1000 in Dako Antibody diluent (S0809, Dako, Glostrup, Denmark), as well as anti-mouse IgG (H + L) and F(ab’)2 Fragment Alexa Fluor® 488 Conjugate (4408S; Cell Signaling, Boston, MA, USA) diluted 1:1000 in Dako Antibody diluent for 1 h. After rinsing in PBS, sections were counterstained with DAPI and a coverslip (9990402, Immuno-mount, Shandon Inc., Pittsburgh, PA, USA).

The intensity of the staining of GZMK-, FasL- and IFN-γ-positive cells was investigated in the placenta (VT and EVT). Additionally, in the placentas the intensity of staining was also investigated in T cells and NK cells. The cell staining process was graded from no expression; + mild expression; ++ moderate expression; +++ strong expression; n no staining (<10% of cells); f focal staining (10–50% of cells); and d diffuse staining (>50% of cells). All the tissue sections were examined using a x40 objective on Olympus BX51 (Olympus, Tokyo, Japan) and photographed with a DP71 camera (Olympus, Tokyo, Japan). Negative control tissue sections were subjected to the same procedure except that they were incubated with PBS instead of primary antibodies. Lymph node tissue was used as a positive control. All sections were analyzed in a blinded manner by two observers (VS and MV).

### 2.4. RNA Isolation and RT-qPCR

A Sigma Aldrich Gen Elute TM FFPE RNA Purification kit was used to isolate RNA from FFPE tissue curls according to the manufacturer’s protocol, as we described previously [4]. Briefly, the concentration of total RNA was measured on a Qubit 4 Fluorometer HS RNA kit. The samples were diluted to match the lowest measured concentration (5 ng/μL). The one-step SYBR® Green RT-qPCR with MMLV and hot-start Taq DNA Polymerase kit (Sigma-Aldrich, St. Louis, MI, USA) was used. The master mix with RNA, selected primers (Table 3), SYBR® Green Taq Ready Mix for Quantitative RT-PCR, Moloney Murine Leukemia Virus Reverse Transcriptase (M-MLV RT) and MgC12 were used and nuclease-free water was incubated in a 96-well plate. All samples were prepared in duplicate, and glyceraldehyde 3-phosphate dehydrogenase (GADPH) was used as the reference gene.

The negative control contained all components of the master mix except the cDNA. The plate was then analyzed using the Applied Biosystems TM 7500 RT-PCR system (Thermo Fisher Scientific, Waltham, MA, USA). Results were expressed as the relative gene expression (relative to the healthy control) as a fold change.

### 2.5. Cytotoxic Assay

Isolation of fresh decidual lymphocytes was performed (Supplementary file S1) according to a modified protocol published by Xu Y et al. [22]. Cytotoxic activity was carried out according to the protocol described by Godoy-Ramirez et al. [23]. The method is based on the simultaneous analysis of conjugate formation between effector (E) and target (T) cells and the analysis of the cytotoxic reaction degree expressed as the proportion of non-viable cells in free (unconjugated) target cells. The distinction between target, effector and conjugated cells was facilitated by the use of the CD45 marker in the combination with the extent of cell granularity. As a smaller number of decidual lymphocytes (effector (E) cells) was obtained from fresh placental tissue, the concentration of effector cells was 1 × 10^6^/mL of viable cells. 

We used K562 cell lines as target (T) cells. These cells originate from patients suffering from erithroleukemia and are extremely sensitive to lysis by NK cells. Cells were grown in a complete RPMI1640 medium with the addition of 20% fetal bovine serum (FCS) at 37 °C and in an atmosphere with 5% carbon dioxide. Two days after feeding, i.e., at a time when the cells are still in the exponential growth phase, the cells were harvested, washed in sterile PBS, counted in a hemocytometer and adjusted to a concentration of 1 × 10^5^/mL in the RPMI1640 medium. 

Effector and target cells were added to polystyrene tubes (12 × 75 mm^2^) with a round bottom in the ratios of 12.5:1 and 50:1 for decidual lymphocytes. Control tubes contained only 100 μL of target or effector cells to which 100 μL of complete medium was added. Control samples were used to exclude non-viable cells, formed spontaneously during the whole night incubation. The tubes were then gently shaken to allow for mixing of the target and effector cells after which they were centrifuged at 200 g for 5 min. This moment is crucial in the preparatory phase of the test since less centrifugal force does not lead to close contact of target and effector cells and thus to effective cytolysis. The cells were then incubated overnight at 37 °C in an atmosphere with 5% of CO_2_. At the end of the incubation, 10 μL of anti-CD45 PE monoclonal antibody and 10 μL of 7-AAD were added to each tube. The tubes were gently shaken and then incubated on ice for 20 min. FACS CANTO II and BD FACSDiva software™ (version 6.0 BD Biosciences, Franklin Lakes, NJ, USA) were used to measure all the cell parameters necessary for the assessment of cytotoxic activity and the degree of conjugate formation. The procedure of passing cells through the cytometer and the analysis followed the instructions described by Godoy-Ramirez et al. [23].

### 2.6. Statistical Analysis

Statistical analysis was carried out using MedCalc software (MedCalc Statistical Software version 12.5.0.0, Mariakerke, Belgium, http://www.medcalc.org. Clinical data were processed using descriptive and inferential statistic methods. Categorical variables were shown as frequencies and percentages. The Kolmogorov–Smirnov test was used for testing the normality of variable distribution. Depending on data distribution, continuous variables were shown as the median and interquartile range (IqR) or as the arithmetic mean and standard deviation by using the Mann–Whitney U-test or T-test for independent samples. Values of ≤0.05 were considered to be significant.

## 3. Results

**Figure 1:**
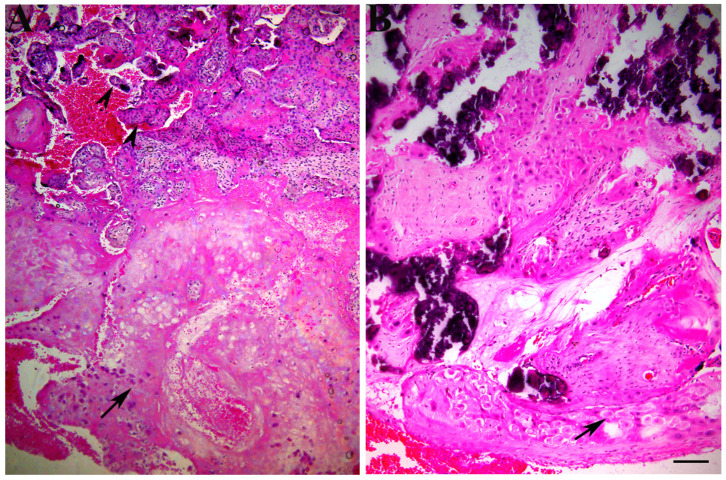
The histological appearance in PE placenta showed (**A**) increased perivillous and intravillous fibrin deposits, hyalinization change, thickened fetal blood capillaries, increased syncytial knots and (**B**) calcification. Extravillous trophoblasts (arrows) and villous trophoblasts (arrowheads). H&E stain. Scale bar—25 μm.

The histological changes in PE placenta included infarcts, increased syncytial knots, intravillous and perivillous fibrin deposits and hyalinization changes that can be seen scattered in the sections (Figure 1).

**Figure 2:**
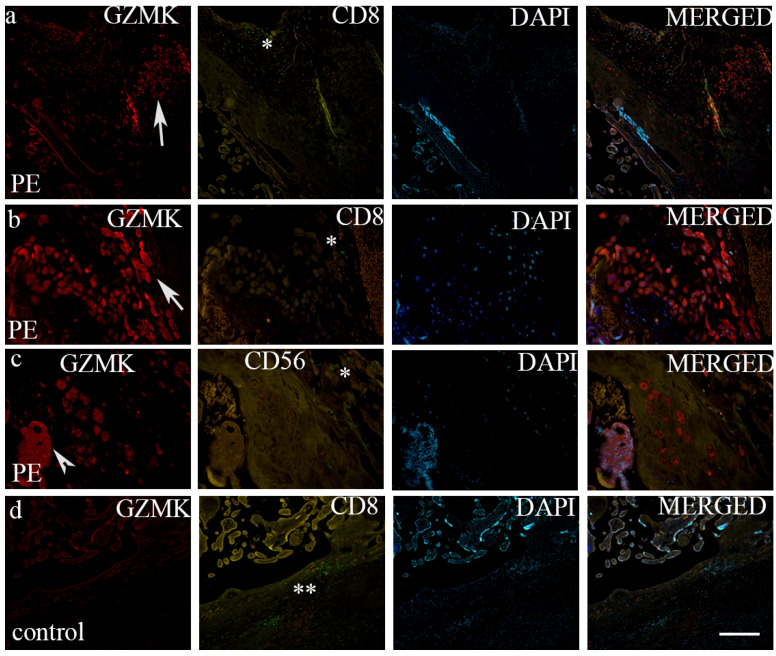
Double immunofluorescence of GZMK and CD8 (**a**,**b**), GZMK and CD56 (**c**) in PE and healthy control placenta (**d**). GZMK-positive cells in extravillous trophoblasts (arrows) and in villous trophoblasts (arrowhead). Scatter CD8 + T cells and CD56-positive cells (NK cells) can be seen in the decidua basalis in PE (*), while abundant CD8+ T cells can be seen in the decidua basalis in healthy control placenta (**). DAPI—nuclear stain; merged—all images in the row. Scale bar—100 μm (**a**,**c**,**d**) and 25 μm (**b**).

**Figure 3:**
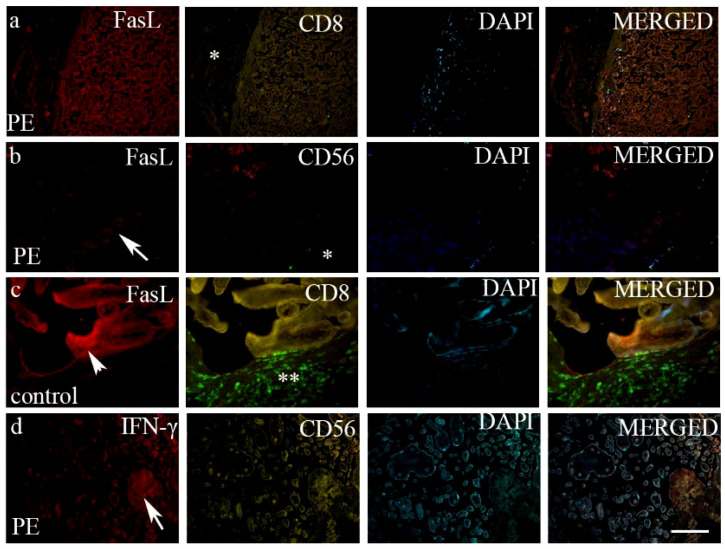
Double immunofluorescence of FasL and CD8 (**a**) as well as FasL and CD56 (**b**) in PE, FasL and CD8 in healthy control placenta (**c**) and IFN-γ and CD56 (**d**) in PE. FasL- and IFN-γ-positive cells in extravillous trophoblasts (arrows) and FasL in villous trophoblasts (arrowhead). Scatter CD8 + T cells and CD56-positive cells (NK cells) can be seen in the decidua basalis in PE (*), while abundant CD8 + T cells can be seen in the decidua basalis of healthy control placenta (**). DAPI—nuclear stain; merged—all images in the row. Scale bar—100 μm (**a**,**d**) and 25 μm (**b**,**c**).

A high variation in the intensity and staining of GZMK- and FasL-positive cells was observed between the control and PE. We observed differences in the intensity and distribution of staining for GZMK, FasL and IFN-γ in preeclampsia and the control placenta. All the investigated markers display no staining or expression in the control tissue. The strong expression of GZMK (Figure 2b) and mild expression of FasL (Figure 3b) were observed in extravillous trophoblasts in the placenta with preeclampsia, while GZMK expression was mild (Figure 2d) and FasL expression was moderate (Figure 3c) in extravillous trophoblast cells in the control placenta. CD8 + T cells and NK cells of the control and PE placenta are displayed in Table 4.

**Figure 4:**
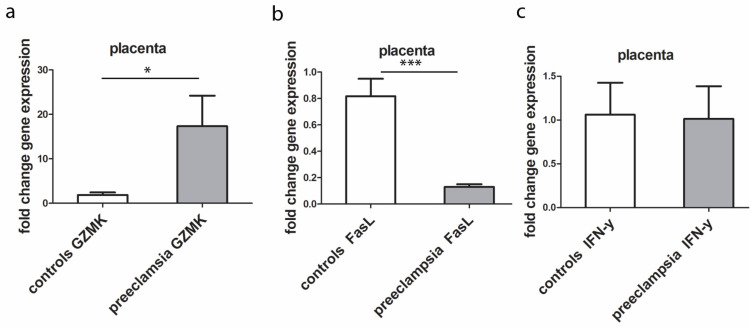
RT-qPCR mRNA fold change of GZMK (**a**), FasL (**b**) and IFN-γ (**c**) from FFPE placental tissue of PE compared to healthy controls. The data were presented as mean ± SD. Mann–Whitney test was used for GZMK and IFN-γ and T-test for FasL; * < 0.01 and *** < 0.0001.

RT-qPCR analysis from FFPE placental tissue confirmed our immunofluorescence results. The expression of mRNA GZMK was statistically higher in PE in comparison to healthy control. On the contrary, there was low expression of mRNA FasL in the PE compared to controls. There was no significant difference for mRNA IFN-γ between PE and control (Figure 4).

The cytotoxic activity of the decidua lymphocytes from patients with PE and the control group were analyzed. The mechanism of the cytotoxicity of NK cells takes place over several steps: (1) adhesion of the NK cell to the target cell, i.e., the formation of a conjugate with K562 (conjugated cells); (2) the recognition of specific ligands (MHC) on the target cells; (3) the activation of the NK cell; (4) the insertion of cytotoxic substances, i.e., (perforin and granzyme) through the membrane of the target cell in order to cause apoptosis or start the apoptosis of the target cell by an alternative way (via the connection of the Fas receptor on the target cells and the Fas ligand expressed on the membrane of NK cells) [24]. Not only do NK cells bind to target cells, but other lymphocytes also do, which in this way prevents the constant contact of NK cells and target cells [25].

**Figure 5:**
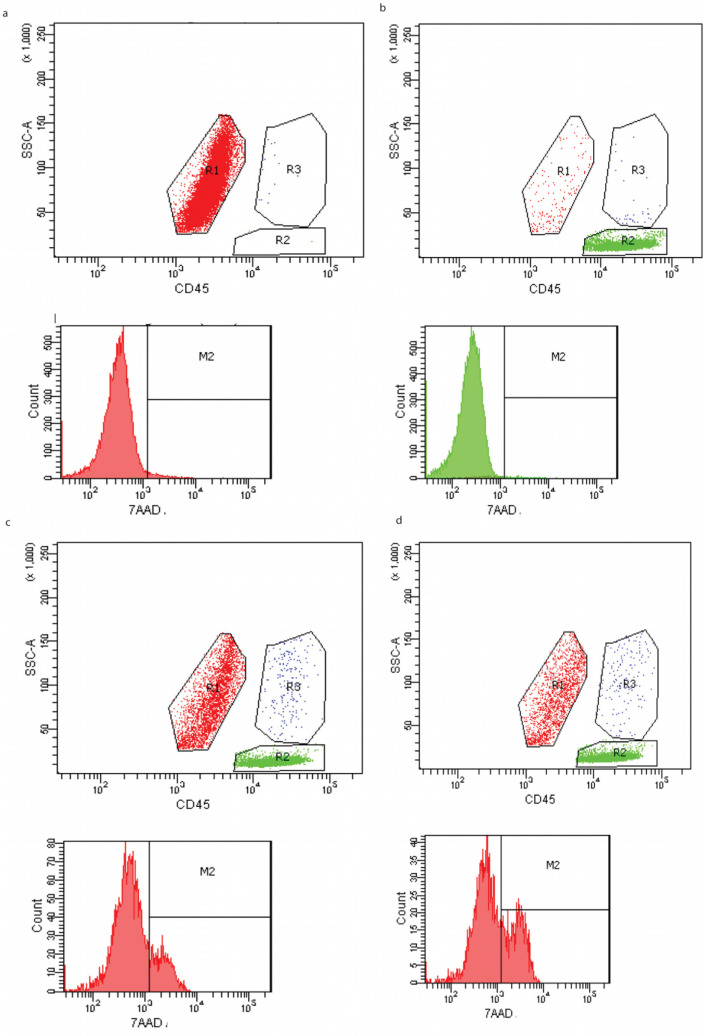
Flow cytometry measurement of cytotoxicity in control samples including only target cells K562 (RED R1) (**a**) or effector cells (GREEN R2) (**b**) using staining with PE-conjugated anti-CD45 and side scatter profiles to identify different cell populations and uptake of 7-AAD to detect cell death (M2). Effector (R2) and target cells (R1) were incubated overnight in mixed cultures according to a ratio of 12.5:1 (**c**) and a mixed culture ratio of 50:1 (**d**), which also display conjugates between targets and the effector, as identified by (R3). The percentage of dead cells in region R1 are calculated from a histogram showing 7-AAD uptake as measured by the intensity of red fluorescence in M2.

**Figure 6:**
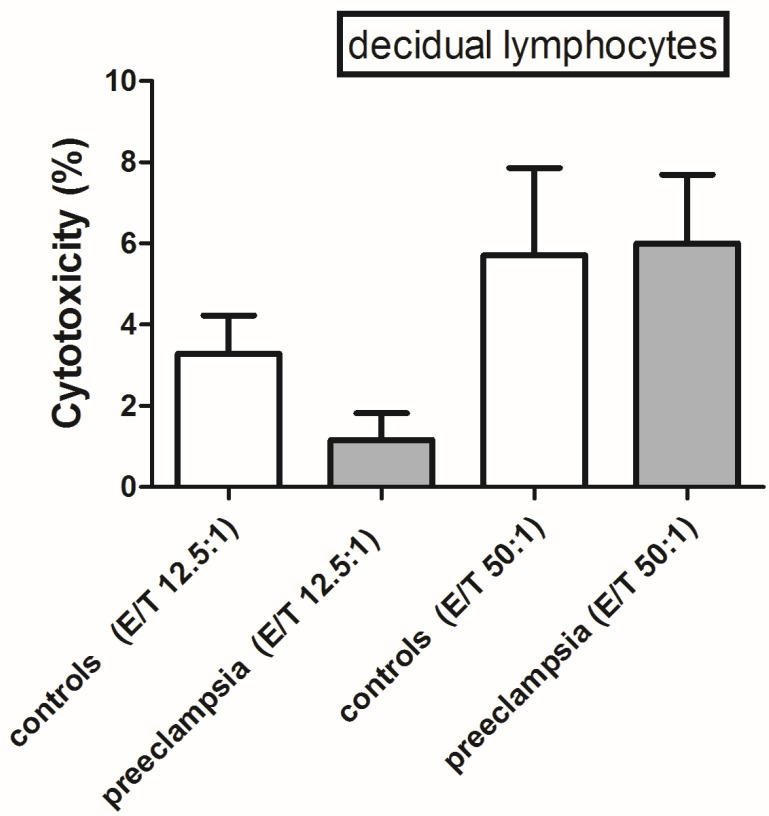
Cytotoxicity of decidual lymphocytes determined from R1 at E/T (effector/target) ratios of 12.5:1 and 50:1.

The distinction between target, effector and conjugated cells was facilitated by the use of the CD45 marker in combination with the extent of cell granularity. The variable and the ratio between effector and target cells (E/T) were examined. The flow cytometry measurement of decidual lymphocytes cytotoxicity in control samples only included target cells K562 or effector cells. We did not observe any cell deaths in the samples (Figure 5a,b). Effector and target cells were mixed in the ratios of 12.5:1, 50:1 for decidual lymphocytes. The level of cytotoxic activity of decidual lymphocytes cells changes depending on the effector and target cell ratio (E/T 12.5:1, vs. 50:1) (Figure 5c,d). However, there were no significant differences between PE and the control (Figure 6).

## 4. Discussion

The role of GZMK in extravillous and villous trophoblasts in the preeclamptic human placenta has not been directly investigated in previous studies. Our study showed that strong GZMK expression was observed in extravillous trophoblasts in the placenta with PE, while GZMK expression was mild in extravillous trophoblast cells in the control placentas. Also, the expression of mRNA GZMK was statistically higher in PE in comparison to that of the healthy control. The study, conducted on rats showed the positive effect of interleukin-17 on GZMK expression, which is significantly elevated in the placenta with PE [26]. Additionally, Travis et al proved that levels of GZMK significantly increased in normal pregnant rats [27]. Increased placental GZMK expression has been observed in cases of placental abruption, leading to severe bleeding during pregnancy [28]. Overall, the findings suggest that GZMK expression in the placenta, particularly in extravillous trophoblasts, may be dysregulated in PE. However, further research is needed to show the specific mechanisms by which GZMK influences trophoblast function and contributes to pregnancy-related complications, as well as its potential as a therapeutic target or biomarker for these conditions.

While FasL and its related pathways have been studied in the context of pregnancy and immune regulation, their specific role in PE is not fully understood. During normal pregnancy, FasL present on trophoblasts induces the apoptosis of Fas-bearing maternal immune cells, while in PE, trophoblasts show increased apoptosis with reduced the expression of FasL [29]. During HELLP syndrome (hemolysis, elevated liver enzymes, thrombocytopenia), the placenta has been reported to serve as the primary source of FasL, which has an impact on inflammation and hypertension during pregnancy and is dysregulated in women with severe PE [30]. Our study also showed that only the mild and moderate expression of FasL was observed in extravillous and villous trophoblasts in the preeclamptic placenta, while FasL expression was moderate and strong in trophoblast cells in the control placenta. Also, there was low FasL mRNA expression in PE compared with that of controls. Another study found that the unbalance of Fas and FasL expressions in the placental syncytiotrophoblast of women with PE may be related to the occurrence and development of the condition [31]. Therefore, our study suggests that Fas ligand levels are altered in PE, with reduced expression compared to that of a normal pregnancy. This finding is in line with a previous study [32].

There is dispute in the literature regarding the role of IFN-γ in pregnant women with PE. Some studies have found that elevated concentrations of IFN-γ in the placenta may lead to PE progression by affecting trophoblast invasion, spiral artery remodeling, trophoblast cell apoptosis and placental angiogenesis [9,33,34,35], while others have found no significant difference in IFN-γ levels between women with and without PE [36,37]. Another study investigating the association between serum levels of cytokines in the first trimester of pregnancy with the onset of PE found that the mean serum level of IFN-γ was significantly higher in the PE group compared to that of the healthy group [38]. In our study, no significant difference was observed between the intensity and extension of staining of IFN-γ in the placenta from controls and PE using the double immunofluorescence method. Also, there was no significant difference in IFN-γ mRNA between the two observed groups. These findings suggest that IFN-γ levels may not be a distinguishing factor between women with and without PE. 

Decidual CD8 + T cells and decidual NK cells play crucial roles in maintaining maternal immune tolerance and placenta development during pregnancy [39,40]. Also, in order to maintain high-quality defense of the organism against various harmful agents, adequate activity of NK and T cells is necessary. Regardless of the recognition mechanism, NK cells efficiently lyse virus-infected, immature hematopoietic or tumor cells [41]. Under cytokine stimulation, NK cells produce and secrete a whole series of cytokines. In direct contact with NK and target cells, cell lysis itself is mediated by a soluble factor that is secreted from the granules into the space between the cells [42]. It comprises a perforin and granzymes, which activate caspases, which lead to programed cell death, i.e., apoptosis [21]. However, the specific role of the cytotoxic activity of decidual lymphocytes in PE is not yet fully understood. During normal pregnancy, decidual lymphocytes constitute 20–30% of the total lymphocytes in the uterus and regulate maternal immune tolerance and placenta development [39]. On the other hand, decidual NK cells are poorly cytolytic and release cytokines/chemokines that induce trophoblast invasion, tissue remodeling, embryonic development and placentation [40]. In pathological pregnancy, such as PE, the dysregulation of various factors, including cytokines, transcription factors, chemokine receptors and microRNAs related to T cells, can lead to the development of PE [43]. Although the cytotoxic activity of decidual lymphocytes varied, there was no detectable difference between PE and controls in the results of our study. In a practical sense, this means that in assessing the patient’s NK activity level, a single finding has no diagnostic weight, but it is necessary to make at least two measurements with an interval of several weeks in order to gain an insight into the dynamics of NK activity. Additionally, if IURG groups could be separated according to ones with and without IUGR, these would significantly improve our precise understanding in this regard. These two issues are lacking in our study and should be taken into account in future studies.

## 5. Conclusions

In summary, our findings suggest that dysregulation of cytotoxic protein expression in extravillous trophoblasts may contribute to the pathogenesis of PE by impairing trophoblast invasion. However, the alterations observed in cytotoxic protein expression did not appear to affect the cytotoxic activity in vitro of decidual lymphocytes. Further research is needed to fully understand the mechanisms underlying these alterations and their implications for pregnancy complications.

## Figures and Tables

**Table 1:** Clinical features of the patients in the study groups.

	Preeclampsia (n = 15)	Control (n = 15)	*p* Value
Maternal age (years), mean ± SD	28.8 ± 3.8	30.7 ± 5.1	0.3130
Gestational age (weeks), mean ± SD	34.8 ± 1.7	34.5 ± 2.1	0.4910
Systolic RR (mmHg), mean ± SD	167.6 ± 14.7	117 ± 11.3	**0.0003**
Diastolic RR (mmHg), mean ± SD	112 ± 11.4	79.5 ± 0.7	**0.0014**
Birth weight (grams), mean ± SD	2015 ± 648.3	2750 ± 282.8	0.6610
Cesarean deliveries (%)	14 (93)	5 (33.3)	**0.0041**
Body mass index (BMI), mean ± SD	22.9 ± 1.9	26.9 ± 4.1	0.0519
Intrauterine growth restriction (IUGR) (%)	11 (73)	1 (6.6)	**0.0191**
Postpartum complications (%)	6 (40)	0 (0)	0.1136
Age of the women	28.50 ± 5.50	29.47 ± 4.26	0.730
Parity (M ± IqR)	0.00 ±1	0.00 ±1	0.928
AST	63.00±8.70	13.58 ± 5.99	**0.026**
ALT	42.17 ± 6.69	16.68 ± 9.79	**0.034**
PLT	229-92 ± 121.00	278.95 ± 68.29	0.052
Leukocyte	10.95 ± 5.02	10.46 ± 2.25	0.921
Hgb	120.92 ± 14.16	115.63 ± 13.31	0.464
Hct	0.35 ± 0.04	0.31 ± 0.09	0.225
Urea	3.62 ± 0.74	4.28 ± 1.96	0.404
Kreatinin	74.83 ± 8.56	78.95 ± 13.89	0.402
Uric acid	336.50 ± 63.25	262.32 ± 70.57	**0.004**
LDH	348.08 ± 167.65	267.16 ± 56.41	**0.028**

Significant *p*-values are depicted in bold.

**Table 2:** Primary antibodies used for double immunofluorescence staining.

Antibody	Dilution	Host	Cellular Localization	Developer
CD8	1:100	Mouse	Membrane	Dako M7103 (Dako, Glostrup, Denmark)
CD56	1:100	Mouse	Membrane	Leica NCL-L-CD56-1B6 (Leica Biosystems Wetzlar, Germany)
GZMK	1:300	Rabbit	Cytoplasm	Sigma Aldrich HPA063181 (St. Louis, MI, USA)
FasL	1:300	Rabbit	Cytoplasm	Sigma Aldrich SAB4501532 (St. Louis, MI, USA)
IFN-γ	1:100	Rabbit	Cytoplasm	Cell signalling 8455 (Cell Signaling, Boston, MA, USA)

**Table 3:** Primers used in RT-qPCR.

Transcript	Forward Primer	Reverse Primer	Amplicon Size	T_m_ F/R	T_A_	CG% F/R
GZMK	TTAAGA CCTTCT GACACC	TGGAA GACA CCTTT ACAG	191	51.1/51.2	51.1	44.44/44.44
IFN-γ	AGCTCT GCATCG TTTTGG GTT	GTTCC ATTAT CCGCT ACAT CTGAA	118	61.5/59.0	59.0	47.62/41.67
FasL	TGGCCT TGTGAT CAAT GAAA	TCATC ATCTT CCCCT CCATC	155	54.5/65.3	54.5	40.0/50.0
GAPDH	ACCCAC TCCTCC ACCTTT GAC	TCCAC CACCC TGTTG CTGTA G	110	64.5/63.9	63.9	57.14/57.14

**Table 4:** Intensity and extension of staining for GZMK, FasL and IFN-γ in placenta from controls and preeclampsia.

		GZMK	FasL	IFN-γ
	CD8 + T cells	+++/f	−/n	−/n
	NK + cells	+++/f	−/n	−/n
Control	Villous trophoblast	+/d	+++/d	+/f
	Extravillous trophoblast	+/d	++/d	+/f
	CD8 + Tcells	+++/f	−/n	−/n
Preeclampsia	NK + cells	+++/f	−/n	−/n
	Villous trophoblast	++/d	++/d	+/f
	Extravillous trophoblast	+++/d	+/d	+/f

−—no expression; +—mild expression; ++—moderate expression; +++—strong expression; n—no staining (<10% of cells); f—focal staining (10–50% of cells); d—diffuse staining (>50% of cells).

## Data Availability

Data are available upon request.

## Supplementary Materials

The following supporting information can be downloaded at: https://www.mdpi.com/article/10.3390/biomedicines12040842/s1, Isolation of decidual lymphocyte from the human maternal-fetal interface. Figure S1: (A) Maternal side of the placenta—visible cotyledons. (B) Thin decidua basalis cleared of villi and fetal blood.
