# Supplementary material for: Effect of Granzyme K, FasL and Interferon-γ Expression in Placentas with Preeclampsia
Source: Biomedicines. 2024 Apr 11;12(4):842. doi: 10.3390/biomedicines12040842 (PMC11048069; doi:10.3390/biomedicines12040842)
Supplement: Supplementary file 1 [file biomedicines-12-00842-s001.zip › biomedicines-2923135-Supplementary.pdf]

Isolation of decidual lymphocyte from the human maternal-fetal interface

Isolation of decidual lymphocyte used for cytotoxicity assay was performed according to a modified protocol published by Xu Y et al.

1. Dissect a fragment of decidua basalis from the surface of one placental cotyledon (Figure 1.A). The tissue fragment should be approximately 1x1 cm in size. The decidua basalis is the part of the placenta that has maternal origin.

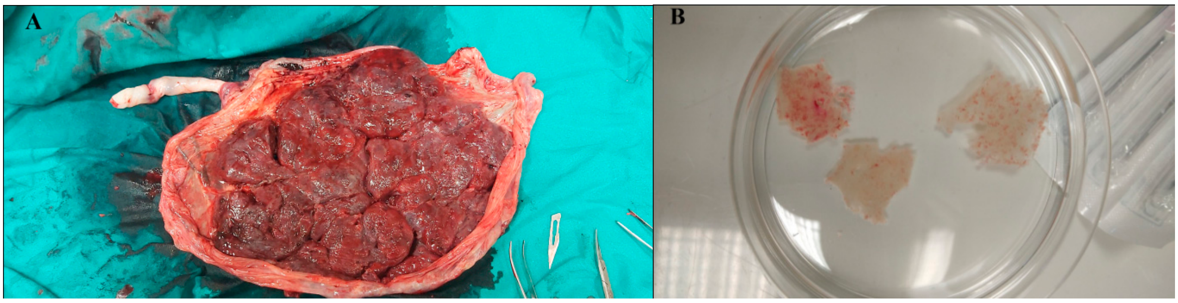

Figure S1. A Maternal side of the placenta – visible cotyledons B. Thin decidua basalis cleared of villi and fetal blood.

2. The fragment of decidua basalis should be as thin as possible and should be placed in a sterile petri dish containing previously prepared sterile 1x PBS (Ca2+ and Mg2+ free).
3. Chorionic villi and fetal blood vessels should be removed by sharp scissors, tweezers and a needle.
4. Wash in 1xPBS to remove blood and achieve a translucent, yellowish-reddish in color decidua basalis.
5. Collect 8 to 12 tissue fragments as described (Figure 1.B).
6. Transfer the tissue samples immersed in PBS from the petri dish into the 50 mL tube and centrifuge at 300g for 5 minutes at room temperature.
7. Remove the supernatant above the tissue pellet without touching the pellet. NOTE: As it contains red blood cells, the pellet is very loose at this point. Add PBS, homogenize with a Pasteur pipette (mix gently) and centrifuge again at 300g, 5 minutes at room temperature.
8. Remove PBS using the Pasteur pipette
9. Put the rest of decidual tissue in a petri dish, and cut it with sharp scissors and a scalpel in order to chop it and homogenize as much as possible. THIS SHOULD NOT BE DONE FOR LONGER THAN 3-5 MINUTES.
10. Transfer the shredded tissue into the empty 15 mL tube and add the acutase pre-heated to 37°C, so that if the volume of pellet is about 3 mL add about 6 mL of acutase, and if the volume is higher than 3 mL, add twice the volume of acutase in relation to the tissue volume.
11. Place the sample on a shaker (at 37°C) where it will gently stirre using acutase which contains proteolytic and collagenolytic enzymes.
12. Centrifuge on 300g for 5 minutes at room temperature, pour out and add PBS to rinse, and centrifuge again on 300g for 5 minutes at room temperature.
13. Remove the supernatant using the Pasteur pipette.
14. Add about 10 to 20 mL of sterile PBS into the shredded tissue and strain through a 100 µm cell strainer into the 50 mL plastic tube. Due to the stickiness of the mixture, more strainers may be used (in our case 2). During squeezing, add the sterile PBS for better rinsing (up to 50 mL of PBS).
15. Centrifuge on 300g for 5 minutes at room temperature.
16. Remove the supernatant above the cells pellet using the Pasteur pipette.
17. Add 15 mL of an ice cold PBS + 2% FBS (cell suspension).
18. Add 15 ml of 20% medium with density gradient - Ficol (1.077 + 0.001 g/ml) to a 50 ml plastic tube and slowly add the cell suspension to the Ficol using the Pasteur pipette in a slightly inclined position.
19. Centrifuge on 800g for 20 minutes at 4^0C without the brake.
20. Lymphocyte will be placed at the interface between density gradient media and PBS.
21. Using the 1000 µL micropipette collect the layer of lymphocyte and put it into the new 15 mL tube. Add 3x higher volume of PBS.
22. Centrifuge on 300g for 5 minutes at room temperature.

23. Remove the supernatant ad add approximately 6 mL of PBS and centrifuge on 300g for 5 minutes at room temperature.
24. Remove the supernatant, add RPMI, determine viability and cell count.
